# Opportunities for Capacity Building to Create Healthy School Communities in the Netherlands: Focus Group Discussions With Dutch Pupils

**DOI:** 10.3389/fpubh.2021.630513

**Published:** 2021-07-29

**Authors:** Bonnie Maria van Dongen, Inge Maria de Vries, Monica Antonia Maria Ridder, Carry Mira Renders, Ingrid Hendrika Margaretha Steenhuis

**Affiliations:** ^1^Department of Health Sciences, Faculty of Science, Amsterdam Public Health Research Institute, Vrije Universiteit Amsterdam, Amsterdam, Netherlands; ^2^Human Movement and Education Division, Windesheim University of Applied Sciences, Zwolle, Netherlands

**Keywords:** building community capacity, community-based approach, pupil participation, health-promoting schools, PA and dietary behaviour

## Abstract

**Background:** To sustainably implement a healthy school community in which stakeholders, including pupils, feel ownership over health-promotion activities, building community capacity is important. Pupils have experiential knowledge that is complementary to professional knowledge, but their perspectives on capacity-building processes are underexposed. This study aims to explore secondary-school pupils' perceptions about key influencers on physical activity and dietary choices and starting points for building community capacity.

**Methods:** Seven focus groups with forty one pupils were held in four secondary schools engaged in a capacity-building intervention. Transcripts were analysed thematically regarding key influencers about choices in the home and school setting and capacity-building strategies (leadership, participation, tailored health-promotion activities and local networks).

**Results:** Parents remained important influencers for making healthy choices, but snacking choices were increasingly made independently from parents based on attractiveness, availability and cost. Choices to engage in physical activity depended on social aspects and opportunities in the physical environment. Pupils considered their influence over the healthy school community limited, desired more involvement, but require this to be facilitated. They identified leaders mainly within formal structures, for example, student councils. They believed health-promotion activities related to the physical environment and project-based activities within the curriculum have the maximum potential to stimulate healthy behaviours in school communities.

**Conclusion:** This study shows that pupils can reflect critically on their physical activity and dietary choices, and on how this can contribute to processes in creating a healthy school community. In order to take an active role, they need to be considered as full partners and leadership roles should be facilitated in existing structures.

## Introduction

Adolescence is an important developmental period in life for promoting healthy physical activity (PA) and dietary behaviours. Healthy behaviours developed during this period often track into adulthood and contribute to reducing the risk of non-communicable diseases later in life (1, 2). However, adolescents tend to engage in more unhealthy behaviours than they did during early childhood (3, 4). For example, they stop playing organised sports and increase sugar-sweetened beverage consumption. Adolescents in lower educational streams are especially at risk of engaging in unhealthy PA and dietary behaviours (5). One common setting to stimulate healthy PA and dietary behaviours among adolescents is their secondary school, because of its pedagogical task and ability to reach this group (6).

An increasing number of schools are taking on their responsibility to promote healthy behaviours by adopting an integral healthy school approach that combines health education with a favourable social and physical environment, school policies on health that include policies on food and PA, and links to the local community and health services (7, 8). One prerequisite for the effective implementation of such an integral approach is that stakeholders, including pupils, feel a sense of ownership over health-promotion activities in their school (9, 10). This can be fostered through meaningful engagement and active participation. Studies have shown that active participation in design, implementation and evaluation processes is a key element in achieving effective and sustainable health-promotion activities (11, 12). Participation by pupils in these processes can enhance their empowerment and increases the likelihood that activities are tailored to their needs.

Although the benefits of pupil participation are widely acknowledged, meaningful participation needs to be facilitated and depends on contextual factors such as the possibilities allowed by extant organisational structures and systems (13). Additionally, it can depend on the attitudes of both pupils themselves and the adults (e.g., teachers and parents) within a school community, and what they consider a desirable level of participation (14, 15). It is therefore important that these stakeholder groups be able to engage in a dialogue about their ideal healthy school and how it can be achieved. To empower stakeholders to take up ownership of such a dialogue and to encourage them to work together, they need to build community capacity (16, 17). This entails the development of knowledge, skills, ownership, leadership, structures, resources and systems at both an individual and an organisational level in order to achieve effective and sustainable health-promotion activities (18).

Previous research has underlined the importance of capacity building for sustainable health promotion in schools (16, 17, 19, 20). Gugglberger and Dür (21) illustrated that schools often understand the importance of the principles of capacity building (e.g., building leadership, structures, resources, etc.), but still find it difficult to institutionalise these principles in day-to-day school processes. In the Netherlands, the FLASH (Fit Lifestyle at School and at Home) intervention has been developed to explore *how* schools can build community capacity for the stimulation of healthy PA and dietary behaviours within the Dutch integral Healthy School (“Gezonde School”) approach (22). This intervention has developed four capacity-building strategies based on methods previously used to enhance community capacity (20, 23): (1) identify leaders within each stakeholder group who inspire others; (2) create a school culture in which stakeholders are able to participate actively; (3) co-design, implement and evaluate tailored activities; and (4) create a network of local partners that can provide continuous resources and partnerships to enable structural embedding. The FLASH intervention puts pupils in a central position and specifically aims to engage those in lower educational streams in capacity-building processes to create a healthy school community. In the Dutch context, this specifically concerns pupils in vocational secondary education, referred to as VMBO^1^ (24).

As research about working strategies for capacity building in a school context is limited (25), it is important to identify opportunities for capacity building as experienced by stakeholders within school communities themselves. Most research about opportunities for capacity building in daily practise focuses on experts in the health-promotion field or on adults within the school community, such as teachers or school leaders (21, 26). This leaves pupils' perspectives underexposed, whereas they should be treated as experts on their own everyday lives, including identifying key influencers in respect of healthy and unhealthy choices they make at school and at home (27, 28). As experts on their own behaviour, they can provide a unique insight into which issues their healthy school community should focus on when stimulating healthy choices for PA and dietary behaviours and how to build capacity in order to create a community that suits their needs.

The goal of this paper is to explore the perceptions of Dutch VMBO pupils about key influencers in respect of PA and dietary choices in their everyday lives, in both the school and the home setting, and to identify starting points for building community capacity in FLASH schools as perceived by these pupils.

## Methods

### Study Design

A qualitative focus-group study was conducted between January and July 2017 among second-year VMBO pupils (average age 13–14 years). Photo-elicitation methodology was used as a launching point to stimulate discussion during focus groups. This method elicits perceptions and stories from participants via non-directive communication, by starting conversations based on photographs participants take beforehand, documenting their own lives (29). Photo-elicitation methods have been shown to facilitate verbalization and in-depth insight, which enhances the quality of results (30, 31). This study falls within the scope of the FLASH intervention and evaluation study. The study protocol of the FLASH intervention has been approved by the Medical Ethics Committee of Amsterdam UMC, VUmc location (reference number 2016.352).

### Setting and FLASH Intervention

This study took place in four secondary schools located in the northeastern region of the Netherlands and engaged in FLASH between September 2016 and July 2019. Each school was provided with the following inputs to facilitate capacity building: hours to appoint a school employee as FLASH coordinator to serve as a leader of the healthy school community, coaching of this coordinator by local public health and educational experts and a start-up budget to implement tailored activities. A more detailed description of this intervention has been provided elsewhere (22). The schools in question varied in terms of such characteristics as number of students, location, contextual factors in the physical environment that might influence PA and dietary choices and the VMBO substreams they offer. Table 1 provides a description of each school.

**Table 1:** Characteristics of FLASH schools.

**School 1: Rural comprehensive (multiple secondary-streams) school**
• All VMBO substreams are offered, but only the “profiles” stream is provided to completion (4 years). For other streams, pupils transfer to different schools after 2 years. In all, 219 pupils were enrolled in the 2016/17 school year; of them, 86 were in the VMBO stream. • Situated in a rural area: few, if any, food outlets within walking distance. • School has a small canteen, operated by ancillary staff employed by the school. This canteen had previously been certified as “Healthy” as part of the Dutch integral Healthy School approach, but as of the start of FLASH that was no longer the case. However, healthy changes made previously were being maintained as much as possible. • PE lessons (indoor and outdoor) are given at a separate location outside the school grounds but within walking distance. The school playground and surroundings offer some opportunities for PA, such as a small football pitch.
**School 2: Urban comprehensive (multiple secondary-streams) school**
• Only the “learning pathways” stream is offered. In all, 711 pupils were enrolled in the 2016/17 school year; of them, 224 were in the VMBO stream. • Situated in a city centre: multiple food outlets within walking distance. • School has a canteen operated by ancillary staff employed by the school. Certification as “Healthy” as part of the Dutch integral Healthy School approach was obtained at the start of FLASH. • School is classified as a school specialising in sports, meaning that it offers more extensive PE than other schools. PE lessons (indoor and outdoor) are given at a separate location outside the school grounds but within cycling distance. The school playground and surroundings offer no opportunities for PA.
**School 3: Urban VMBO-only school**
• Offers only the “profiles” substream. In all, 520 pupils were enrolled in the 2016/17 school year. • Situated in a city centre: multiple food outlets within walking distance. • School has a canteen operated by ancillary staff employed by the school. Certification as “Healthy” as part of the Dutch integral Healthy School approach had not been obtained at the start of FLASH. • PE lessons are given within the school grounds. The school playground and surroundings offer no additional opportunities for PA.
**School 4: Rural VMBO-only school**
• Offers only the “profiles” substream. In all, 228 pupils were enrolled in the 2016/17 school year. • Situated in a rural area: few food outlets within walking distance. • School has a canteen operated by a commercial caterer. This school had only recently opened at the start of FLASH and certification as “Healthy” as part of the Dutch integral Healthy School approach had not yet been obtained. • PE lessons are given within the school grounds and at an off-site location within cycling distance. The school playground and surroundings offer no additional opportunities for PA.

### Procedure

In order to make initial acquaintance with the topic “making choices in PA and dietary behaviours,” second-year VMBO pupils engaged in a classroom assignment in which they took and discussed photographs of situations in their daily life where they made PA or dietary choices. This classroom assignment was conducted between February and April 2017. Following this assignment, pupils were invited to join an in-depth focus group to further explore key influencers in respect of PA and dietary choices and what they mean in creating a healthy school community. Recruitment was done by the FLASH coordinator, due to their familiarity with the pupils concerned, and was based on willingness to participate. As only a limited number of pupils responded to this call of the FLASH coordinator to the entire second-year VMBO stream, the coordinator purposively recruited additional participants from within this cohort, with a view to achieving fair representation by gender and by the VMBO substreams offered by the school. Pupils were excluded if they were unavailable to attend the focus group during school hours. Written informed consent was obtained from all participants, as well as from a parent or guardian if they were younger than 16.

In June–July 2017, two focus groups were held at schools 1, 2, and 3, and one at school 4. Each group consisted of between three and eight pupils. As preparation, participants were asked to engage in a new and more structured photo assignment. A week before the focus group, they received instructions from the researcher. They were asked to take photographs of five situations they encountered in their daily lives: (1) an easy choice and (2) a hard choice regarding dietary behaviours; (3) an easy choice and (4) a hard choice regarding physical activity; and (5) something else important to them about PA or dietary behaviours. Pupils were instructed to send photos to the researcher, accompanied by a descriptive caption. They received a reminder approximately 2 days before their focus group. Two researchers grouped photos based on similar behavioural choices and formulated discussion statements. These statements generalised the grouped photos and represented the opinions and issues of pupils at that specific school.

### Data Collection

Two researchers trained in qualitative research were present during all focus groups: the first two focus groups were guided by BD and MR, and other focus groups were guided by BD and a trained research assistant. BD led the discussion and the other researcher observed and took field notes. Each session was divided into two parts. Pupils were first invited to discuss key influencers with regard to PA and dietary choices that they find important when creating a healthy school community. The interview guide for this part of the focus group was based on the socio-ecological model, which highlights individual, social, organisational, and community influences on health behaviour (32). The discussion statements and a selection of photos that were in agreement or in contrast with each of the statements were used as a launching point for conversation. At each school, three or four statements were presented; these varied from school to school as their photos also differed. For example: “I prefer foods that are fast and easy to prepare, regardless of whether I am home or at school” or “I like exercise most when I can play with friends.” Pupils were invited to react to these statements, and then asked why they agreed or disagreed and to provide reasons for their behavioural choices. They were encouraged to discuss their opinions of statements with each other. If a conversation did not start spontaneously, pupils were asked questions such as “Who recognises him/herself in this statement?” and “If it is your photo accompanying the statement, can you explain why you took it?” During each focus group, researchers asked about individual choices and how the home or school setting played a role in them.

The second part of the session focused on what the ideal healthy school community was and how this could be achieved. Pupils were then asked to write down ideas about changes to the school setting that would stimulate healthy PA and dietary behaviours. Here, they were encouraged to take into account what they had discussed in the first part of the focus group. The interview guide for this part was based on strategies for capacity building that are central in the FLASH intervention, namely leadership, participatory school culture, tailored health-promotion activities, and local networks (22, 23). Each pupil was given the opportunity to share their ideas for their ideal healthy school community, which were then discussed. Starting points for capacity building were identified by asking questions such as “What does school need to do to make this happen?”, “What role can you and your fellow pupils play?” and “Who else can help realise this idea?” At the end of the focus groups, participants were each given a small reward.

### Data Analysis

All the focus groups were audio recorded and transcribed verbatim. Maxqda 2018 software was used to analyse transcripts. Two members of the research team (BD and IV) independently analysed the first two transcripts applying open thematic coding techniques. This resulted in the identification of key influencers with regard to PA and dietary behaviours that pupils felt were important to take into account when creating a healthy school community. In order to categorise codes into themes, the socio-ecological model (32) was used to differentiate between personal influencers and those in the social and physical environment around pupils. Additionally, differences between dietary behaviour, PA and health in general were examined. The coding by the two analysts was compared and discrepancies were resolved through discussions with a third member of the research team (CR). All manuscripts were coded with the finalised coding system by the two analysts, who met regularly to discuss the organisation of themes and subthemes. The photos taken by participants to capture moments when they made PA or dietary choices were not used during this analysis, since they served only as conversation starters and were not meant to stand by themselves. Examples of photos can be found in Appendix I.

The two analysts then coded all manuscripts with the aim of identifying starting points for building community capacity based on pupils' perceptions. An inductive approach was used, where segments related to capacity building strategies central in FLASH were coded and grouped to develop themes for capacity-building opportunities (22, 23). Codes in these strategies were defined as follows: (1) leadership: which people (adults and pupils) are leaders and how can leadership improve; (2) participatory school culture: degree to which pupils are able to provide input and how participation can improve; (3) tailored health-promotion activities: perceptions about current health-promotion activities and how (new) activities can improve; and (4) local networks: influences around school and how to potentially deal with this. The analysts met regularly to discuss similarities and discrepancies. All final results were discussed by the entire research team.

## Results

In total, 19 male and 22 female pupils with a mean age of 13.9 years (range 12–16) participated across seven focus groups (Table 2). On average, focus groups lasted 75 min (range 64–86). Table 3 provides an overview of the results.

**Table 2:** Pupil characterics per school (*n* = 41).

**School (total n second-year VMBO pupils)**	**Participants in focus group 1**		**Participants in focus group 2**		**Total *N* per school**	**Average age per school (SD)**
	**M**	**F**	**M**	**F**		
School 1 (*N* = 66)	4	3	–	3	**10**	13.8 (0.63)
School 2 (*N* = 72)	6	2	3	3	**14**	14.0 (0.55)
School 3 (*N* = 80)	–	5	3	4	**11**	13.6 (1.16)
School 4 (*N* = 56)	3	3	NA^a^	NA^a^	**6**	13.8 (0.75)

a *Not applicable*.

**Table 3:** Overview of topics discussed and key points based on pupils' perceptions.

**1. Behavioural choices that influence creating a healthy school community**		
**Topics discussed about dietary choices**	**Key points based on pupils' perceptions**	**Sample quote**
Making more independent choices with regard to snacking behaviour	- You only snack on what you find tasty: mostly unhealthy food, but fruit can also be tasty. - Snacking in school is influenced by what is offered in the school canteen: healthy products are not attractive to buy due to price and quality. - Local retailers offer cheap and unhealthy snacks.	- “Things that are unhealthy, you pick them more easily because they're tastier. Things that are healthy are not really tasty.” – F, School 3, group 1 - “They only have unattractive apples. They look like they're not supposed to be sold any more in the supermarket and so school got them to give away.” – F, school 2, group 2
Making more independent choices with regard to eating breakfast	Eating breakfast is important, but it is easier to skip breakfast.	“Of course you can eat breakfast, but most of the time you don't have enough time if you have to go to school. I'm not getting up any earlier than I have to.” - M, school 4, group 1
**Topics discussed about PA choices**	**Key points based on pupils' perceptions**	**Sample quote**
Many choices have become habit or routine.	You don't think about exercise when you go to training every week or take your bike to go everywhere: you just do it.	“I always have to go to practise on Tuesday and yeah. I just go. Even when the weather is bad or something.” – M, school 2, group 1
Factors that are influencers of a conscious PA choice.	- Exercise means that you can hang out with friends, which is fun. - Sport can be a stress-reliever.	- “For me, exercise is not about losing weight – it's about the people who are there, because they make it fun.” – F, school 2, group 2 - “I think that exercise for most people is also a stress-reliever: you can get rid of some energy you can't get rid of at school.” – M, school 2, group 1
The role of PE in school.	- It is good that school offers PE, because that makes sure you stay physically active. - PE needs to be fun in order to actively participate.	- “PE makes sure that everyone gets at least some exercise.” – F, school 1, group 2 - “A while back we played an active game during PE: that was fun, so I actively participated. But if we do baseball for five lessons, I don't enjoy it any more so I do less.” – M, school 2, group 2
The physical environment can influence how active pupils are on school days.	What aspects of the environment were identified varied by school.	“We have a small football pitch in the playground that no-one uses. I'd prefer a proper pitch next to school, to be active during breaks.” – M, school 1, group 1.
**Topics discussed about health in general**	**Key points based on pupils' perceptions**	**Sample quote**
Views on health in general.	Health is important, but not a deciding factor for behaviour.	• Researcher: “What do you all think about health?” • Multiple pupils: “Important!” • Researcher: “Does it play a role in choices you make?” • Pupil: “No, it's something I think about later on.” - M/F, school 3, group 2
Social influence of parents on choices.	Parents are responsible for making the healthy choice.	On healthy eating: “My parents have to tell me to eat my vegetables at dinner. Otherwise I won't.” – M, school 1, group 1
**2. Starting points for building community capacity**		
**Discussed strategy**	**Key points based on pupils' perceptions**	**Sample quote**
Leadership.	- Official structures were identified as leadership opportunities. - For pupils, the student council is an important structure. - The way the student council works can be improved.	- “I don't think I can play a role. That's up to the head teacher.” – F, school 1, group 1 - “I'm on the student council, so we can arrange things.” – F, school 3, group 1 - “We need ideas from other pupils, but they don't submit them. We've tried a lot of ways, such as a letterbox in different places, but so far nothing has worked.” – F, school 1, group 2
Participatory school culture.	- Pupils feel they have limited influence over what happens in their healthy school, except when they come up with concrete ideas.	- “Well, school likes to be in charge. They do look at what we need, but also at what's practical for them. And most of the time if it costs too much, there's nothing we can do.” – F, school 2, group 2
	- Suggestions to have more input at a practical level relate to helping in the canteen and having a say during PE.	- “A few pupils could help to make smoothies from old fruit that no-one would buy any more.” – F, school 3, group 1
Ideas for health-promotion activities.	- Change the physical environment to make the healthy choice more attractive, i.e. the canteen for nutrition and the playground for PA. - Create projects that can become part of the curriculum and include a challenging aspect.	- “The canteen should offer cheaper healthier products, like bowls of assorted pre-sliced fruits. That's tasty and healthy!” – M, school 4, group 1 - “A while back we did a project with fruit and vegetables where we had to make up our own recipe. We could make that into a snack-battle project: the best recipe wins a prize and is sold in the canteen.” - M, school 3, group 2
Local networks.	- Limited ideas for local support, but pupils acknowledge easy access to unhealthy and cheap snacks around school. - Rules against leaving school premises are seen as ineffective to combat this issue.	- “A lot of kids go to the supermarket or McDonald's during break times. They're very close by.” - F, school 3, group 1 - “We're not allowed to leave the premises during school hours, but if we go to PE at a different location then we pass the supermarket. I usually stop and buy something then.” – M, school 2, group 1

### Behavioural Choices That Influence Creating a Healthy School Community

#### Perceptions Concerning Dietary Choices

Pupils stated that they especially made independent choices with regard to snacking behaviour and eating breakfast. Fruit was considered an easy healthy snack, but pupils said they only picked fruit that they found tasty and easy to eat. However, they also mentioned that they often preferred unhealthy options, such as sweets or savoury snacks. They said that they did not buy fruit from local retailers because their friends also did not: “*I go with my friend to [local retailer], but not to buy fruit. I buy snacks or candy!”- M, school 2, group 2*.

At all the schools, pupils identified the school canteen and local retailers as the main providers of snacks. With regard to buying healthy snacks in the canteen, they complained that prices were too high compared to the quality they received. At schools 2 and 3, pupils considered their canteen to be healthy but mentioned easy access to retailers close by and said they often bought cheap snacks there. Pupils of schools 1 and 4 were critical as to whether their canteen was truly healthy, because they questioned the information about the healthiness of products: *When they told us we were going to move to this building, they said it would also be a healthy school. But if I look in the school canteen, it does not seem healthy at all” – M, school 4, group 1*.

As with snacking behaviour, pupils reported making more independent choices from parents around breakfast habits. They mentioned a gap between knowing the healthy choice and acting upon it. Reasons they provided for skipping breakfast were not feeling hungry or giving priority to sleeping in.

#### Perceptions Concerning PA Choices

Pupils reported that many choices they made around PA had become habits. They remarked that daily travel by bicycle results in more PA as it is considered normal to take their bike to school. At school 3, one pupil noticed that many pupils used inactive ways of transport to reach school, such as bus or car: “*A lot of people here do not come by bike, but instead take the bus or are dropped off by their parents. Even some people that live close by!” – M, school 3, group 2*.

If pupils reported making a conscious choice to be physically active, their main incentive was the social aspect. They also mentioned that sports club membership drops with age, and because of that they emphasised the importance of offering physical education at school. Pupils added that PE lessons need to be fun in order for them to participate actively, and that this depended on the teacher and the variety of activities offered.

Finally, pupils talked about the influence of the physical environment on how active they were during a school day. They focused on different aspects of this environment. For example, pupils at school 1 highlighted the limited exercise options around the school, while those at school 4 focused on the stairs in their school building: “*Our building has such steep stairs that we have to walk a lot: totally not fun.” – F, school 4, group 1*. Overall, pupils felt that the physical environment of their schools did not challenge them to be active.

#### Perceptions Concerning Health in General

At all the schools, pupils considered health to be important but not the deciding factor in their behavioural choices. They mentioned that parents were the main influencers when it comes to making healthy choices, in both PA and dietary behaviours: “*My mother told me to go out running instead of playing games on my computer.” – M, school 2, group 2*.

### Starting Points for Building Community Capacity

#### Strategy 1: Leadership

Pupils pointed primarily towards formal leadership structures with regard to potential leaders for the healthy school community. Examples they mentioned were head teachers, school managers or the parent council: “*The easiest way is to go to the parent council” – M, school 1, group 1*. Pupils did not immediately see a role for themselves personally, but did point to the student council. At schools 1, 2, and 3, some participants said there was a student council in place, but not all participants were aware of this: “*Researcher: who among pupils can help? Pupil 1: we do not have a pupil team. Pupil 2: yeah, we do have a pupil council right or not? Pupil 3: Yes, we do. My friend is a part of that” – M/F, school 2, group 2*. Pupils therefore suggested that the council needed to become more visible. For example, by publicly choosing representatives during mandatory guidance hours. Additionally, participants who were members of the student council stated that they needed more input from fellow pupils on what potentially can be changed in the healthy school community in order for them to take a leadership role. Overall, pupils agreed that becoming an active member of a healthy school community should be rewarded or made easy: “*I would not mind helping if I could leave class ten minutes earlier. But I do not think I would do it if it means I would have spent two hours after school on it” – F, school 2, group 2*.

#### Strategy 2: Participatory School Culture

Pupils considered their influence within the healthy school community to be limited. Some indicated that they found it difficult to express their opinion to official leaders, others stated that they were taken seriously if they came up with concrete and manageable ideas: “*We suggested they put up a basketball net in the school yard and the school leader told us he is going to make that happen over the summer” – F, school 1, group 2*. Pupils made suggestions about how they could be involved with the healthy school community at a practical level. One example that was often mentioned was helping in the school canteen by making healthy sandwiches or smoothies. Pupils also felt that PE lessons could be made more enjoyable if mandatory subjects were offered in a game set-up where the focus is on fun and play in contrast of learning a skill, because they believed this offered more opportunities to have a say during these lessons and for social interaction.

#### Strategy 3: Designing and Implementing Activities

Health-promotion activities that pupils thought had the greatest potential often related to the physical environment. With regard to dietary behaviour, they suggested making healthy options in school canteens more attractive. These options should be easy, such as offering multiple varieties of pre-sliced fruit, and at prices comparable to local retailers. With regard to PA, pupils suggested improving their school playgrounds. They discussed how spaces should look or change in order to make playing sports possible. Implementation issues were also discussed: “*Pupil 1: The school playground could be improved if they would add multiple options, like those courts where you can play soccer and basketball. That way we can choose. Pupil 2: But then there would be arguments who gets to play when, because there are two schools in this building who use the school playground. Pupil 3: Couldn't we just make rules, like on this day we get that space and on another day other people?” M/F, school 4, group 1*. Additionally, pupils proposed project-based activities that could become part of the regular curriculum. These suggestions related mainly to informed choices concerning dietary behaviour. Pupils considered it important to learn practical skills they can apply in their own personal lives, such as cooking. They also said they would be motivated by projects that involve a challenge and offer a reward for the effort they put in.

#### Strategy 4: Creating Local Networks

Pupils found it difficult to answer questions about who could help the school to create a healthy school community. However, many indicated that there were multiple food retailers offering cheap and unhealthy snacks in the vicinity of schools: “*There is a cheap supermarket close by so I can easily go there during school hours. Even if the price in the canteen is similar, I go there because I get a bigger portion” – F, school 3, group 1*. Rules against leaving the premises during school hours were seen as ineffective, because they could easily be circumvented. Additionally, pupils at all the schools saw opportunities to create a more active environment by repurposing unused land around their school.

## Discussion

The goal of this paper was to explore the perceptions of VMBO pupils about key influencers in respect of PA and dietary choices in everyday life and to identify starting points for building community capacity in schools implementing the FLASH intervention. Pupils indicated that parents are still very important influencers in respect to their PA and dietary behaviours. But especially in respect of snacking behaviour, they increasingly seemed to be making their own choices. Similar to determinants of food choices in general (33), snack choices depended on the attractiveness, availability and cost– resulting in either healthy choices (fruit) or unhealthy ones (sweets). Additionally, the accessibility to food retailers around the school that offer cheap and unhealthy snacks proved to be an important influencer. Physical activity was often reported as being habitual, such as cycling to school, or being organised, such as PE lessons or sports club membership. The choice to engage in PA depended on having fun, being with friends and the opportunities available in the physical environment.

Overall, pupils considered health important but reported that their attitude towards it did not influence their PA or dietary choices. In line with previous research (4, 33–35), they assigned responsibility for making healthy choices primarily to parents and to stakeholders at school: they feel these actors should create a physical and social environment that facilitates healthy choices at home and at school. One potential reason why pupils were hardly concerned with their own healthy behaviour is that making these choices does not have immediate consequences for their health. Adolescent development is characterised by a limited ability to oversee long-term consequences of unhealthy choices (36, 37). The pupils themselves expressed that they can behave unhealthily as long as they feel healthy (4).

Although pupils were hardly interested in consciously changing their own PA and dietary behaviours, they did indicate that they were interested in being involved in creating a healthy school community: not only by participating in activities, but also by acting as partners in the process of creating such a community. Within this process of participation, they particularly emphasised to enjoy the social aspect of collaboration in contrast to focussing on their own health behaviours. Pupils provided suggestions on how a healthy school community could be built and what their own role in this process could be.

### Identifying Leaders and Creating a Participatory School Culture

Pupils identified leaders primarily within formal structures of their school communities, such as head teachers and parent councils. As a result of these hierarchical leadership structures, pupils' perceptions seemed to indicate that none of the FLASH schools currently has a highly participatory culture (14). They expressed a desire to be involved, but seemed unsure how far their influence could reach and how they could become leaders in creating healthy school communities.

Previous studies have found that pupil engagement is an important implementation factor for sustainable health-promotion in a school setting (10). Similar to pupils in this study, Mäkelä et al. (38) showed that pupils can be full partners in designing the educational environment. Pupils' experiential knowledge can complement professional knowledge, because new perspectives may evolve from this exchange (39, 40) and thus can improve ownership and sense of empowerment of pupils (28, 41). However, pupil engagement and appointing peer leaders occur mostly when facilitated by teachers or researchers (42, 43). This was also evident in the present study, as pupils identified student councils as an opportunity for leadership and participation but concluded that more support is necessary to ensure that these bodies do actually represent the student voice.

The value of student councils in representing the student voice when it comes to creating healthy school communities has been emphasised in previous research (44, 45), because these bodies already connect with daily practise in schools (46). It is important, however, to consider the conditions necessary for genuine pupil participation. Alderson (44) has shown that ineffective councils, such as token ones with no real decision-making power, can counteract a participatory school culture. Conditions mentioned by pupils during this study were in line with those previously identified as needed for effective councils, such as visible procedures to choose representatives, regular and clear communication with other leaders in the community and decision-making powers (45).

### Designing Activities and Creating Local Networks

Pupils indicated that they wanted to be involved in designing health-promotion activities within the school community. During this study they showed that they were able to generate ideas once engaged intellectually with their own and their peers' healthy or unhealthy choices. In relation to the implementation of activities, moreover, they were able to oversee consequences, such as the need for behavioural rules. This illustrates that pupils, if provided with the opportunity to do so, are able to add their experiential knowledge of daily school life to theory about health promotion and behavioural change in schools. Furthermore, their suggestions are in line with promising health promotion strategies, such as focus on experiential activities and involvement of parents (47).

Health-promotion activities proposed by pupils were consistent with key influencers they mentioned with regard to their behavioural choices. For example, every focus group discussed the potential of the school canteen in promoting healthy snack choices as a solution to pupils' perceptions that accessibility influences their choices. Similar to other studies (35, 48), pupils questioned whether their school canteens currently nudged them to healthier choices. In the Netherlands, schools have no tradition of offering school meals. Most students bring their lunch from home and buy additional products at school canteens or food retailers around the school. Although a lot of attention is currently being paid in the Netherlands to the implementation of guidelines for healthy school canteens (49), pupil involvement in improving these canteens in line with their needs and wants is not yet common. Previous research underlines this as a promising strategy to optimise healthy canteens (50, 51). Suggestions made by pupils during this study highlighted the use of nudging strategies (52) (making healthy options more attractive and easily accessible) and an integral approach (linking educational activities to the food environment at school, including canteens). Although pupils only mentioned local food retailers as a key influencer and not a suggestion for strengthening the schools' local network, it is also important to view the school as part of a wider system as previous research supports the notion that the environment around schools in the Netherlands can be seen as obesogenic (53, 54). Suggestions with regard to PA were also consistent with key influencers, such as redesigning the physical environment around the school to make it easier for pupils to engage in organised and fun activities during breaks. In order to implement these suggestions by pupils, schools will need to connect with stakeholders outside the school system (e.g., policymakers and local retailers). This process needs to be facilitated by leaders of the healthy school community as well as leaders outside the school system (55, 56).

### Strengths and Limitations

One strength of this study is its use of photo-elicitation methodology and thus including pupils in a meaningful way as actors of their own choices. VMBO pupils, which are pupils that are engaged in the vocational educational levels in the Netherlands, seemed to especially benefit from expressing themselves in a visual manner. Classroom sessions provided us with insights into how this method could be applied within the study's population of VMBO pupils and within the setting of the participating schools. In particular, it showed us that pupils at this level need specifically formulated assignments and guidance. We therefore added discussion statements to the focus-group protocol that helped pupils to start the conversation, but—since photo-elicitation methodology puts participants in the lead—also gave them the freedom to digress to other topics they wanted to discuss.

The included schools in this study varied in terms of size, pupil population, location and contextual factors in the physical environment, but the number of schools and pupils were quite small. Moreover, they participated on voluntary basis, which could have led to a selection bias towards motivated pupils. Therefore, the findings of these focus groups might not be applicable to other secondary schools in a different context with a different population. Nevertheless, this study provides important insights into young teenagers' perceptions about their health choices and how they can be involved in thinking about ways to meaningfully stimulate healthy behaviour at school. Pupils seemed to know what is healthy and what is not, and can suggest ways of changing existing barriers. Given that the purpose of this study was to identify starting points for building community capacity from pupils' perspectives, the number of pupils taking part was considered sufficient, because it did reveal opportunities to actively involve pupils in the process of creating healthy school communities. Because of the small number of schools and pupils, it is difficult to say if data saturation on pupils' perceptions was reached. However, subjects that pupils discussed on key influencers and on capacity-building opportunities were comparable across all focus groups, suggesting data saturation was reached.

### Implications for Research and Practise

There is currently limited research into the part pupils in vocational secondary education can play in creating healthy school communities. For example, how they can take up leadership roles, be engaged with the various processes involved—from designing activities to evaluating them—and feel ownership over their healthy school community in day-to-day practise. Previous research shows that active pupil participation is important for effective health promotion and has the potential to positively influence the school organisation and the individual pupil (10). However, there is limited insight into how pupils can be involved in healthy school communities. This study provides starting points for how that can be done in respect of stimulating healthy PA and dietary behaviours. Further research is needed to reveal how schools in different contexts can achieve participation throughout their pupil population. In addition to the current study among pupils, in which we specifically used photo-elicitation to enable this target group to voice their perceptions, we will also investigate perceptions of other stakeholders in FLASH, such as school personnel and parents, on how to build community capacity by means of interviews (22).

Additionally, we have found that photo-elicitation is not only a suitable tool for gaining insights into pupils' perceptions, but also that it has the potential to empower pupils within the process of creating healthy school communities. However, more research is needed on how photo-elicitation can be used in the day-to-day practise of a school setting. Studies in this respect should focus on if and how this methodology can be embedded in a schools' curriculum so that more pupils from different backgrounds and ages can be involved. In our experience, VMBO pupils are willing to participate actively if photo-elicitation is applied to a concrete topic within the healthy school community, for example, redesigning the physical environment. Photo-elicitation methodology has previously shown to be flexible in its' implementation and can be adjusted to also suit different contexts', cultures, resources and health topics (57–59). For follow-up research it would be interesting to find out how perceptions differ from other age groups and areas using this methodology.

Because photo-elicitation methodology enables freedom among participants and flexibility in how the method is applied, findings in these types of studies are mostly context-specific to organisations and structures. However, overarching findings such as the pragmatic suggestions pupils gave on improving leadership and participation structures in student councils and school canteens, can be considered relevant even in schools or communities that do not have the same profile as the schools in this study. As is similar with other context-specific studies, replication opportunities lie in other school communities applying photo-elicitation in a similar manner to start a conversation with their pupils on how to tailor these opportunities to their own context (60).

## Conclusion

Pupils in vocational secondary education have shown in this study that they can reflect critically on their own PA and dietary choices, the health implications of those choices and factors that influence them. They indicate that health-promotion activities should focus on stimulating healthy snacking behaviour and increasing enjoyable PA opportunities in the school setting, both as part of the curriculum and within the physical environment. Pupils are open to and want to contribute to the process of creating a healthy school community. In order to assume an active role, however, they need to be considered as full partners and leadership roles need to be facilitated within existing structures. It is also important to support and guide pupils in taking up this role. As pupils indicate that parents and local retailers are important influencers with regard to health behaviours, in addition it is crucial that the home setting and local networks be involved when building community capacity and creating healthy school communities.

## Data Availability Statement

The raw data supporting the conclusions of this article will be made available by the authors upon reasonable request, without undue reservation.

## Ethics Statement

The FLASH intervention and the studies involving human participants were reviewed and approved by Medical Ethics Committee of Amsterdam UMC, VUmc location (reference number 2016.352). Written informed consent to participate in this study was obtained from participants and the participants' legal guardian/next of kin if they were younger than 16.

## Author Contributions

All authors approved the manuscript for submission and have participated in the concept and design and drafting or revising of the manuscript.

## Conflict of Interest

The authors declare that the research was conducted in the absence of any commercial or financial relationships that could be construed as a potential conflict of interest.

## Publisher's Note

All claims expressed in this article are solely those of the authors and do not necessarily represent those of their affiliated organizations, or those of the publisher, the editors and the reviewers. Any product that may be evaluated in this article, or claim that may be made by its manufacturer, is not guaranteed or endorsed by the publisher.

## Footnotes

^1^**The Dutch education system:** Pupils entering secondary education in the Netherlands are streamed by aptitude into one of four forms of schooling: practical (PrO), vocational (VMBO), professional (HAVO) and pre-university (VWO). About 60 per cent of pupils join the VMBO stream, a four-year programme (ages 12-16) intended as a route into post-secondary vocational training (MBO). After a two-year common basic curriculum, the VMBO trajectory itself splits into a number of substreams with selection by ability, interests and ambitions: “learning pathways” that focus on theory and “profiles” that focus on practice. Individual schools often provide more than one form of schooling, e.g. comprehensive schools (PrO/VMBO/HAVO/VWO), and multiple VMBO substreams.

## Abbreviations

PA: physical activity
FLASH intervention: fit lifestyle at school and at home intervention.
