# Supplementary figures and images for: Opportunities for Capacity Building to Create Healthy School Communities in the Netherlands: Focus Group Discussions With Dutch Pupils
Source: Front Public Health. 2021 Jul 29;9:630513. doi: 10.3389/fpubh.2021.630513 (PMC8358072; doi:10.3389/fpubh.2021.630513)

**Appendix I – examples of photos taken by pupils**

**School 1**

**
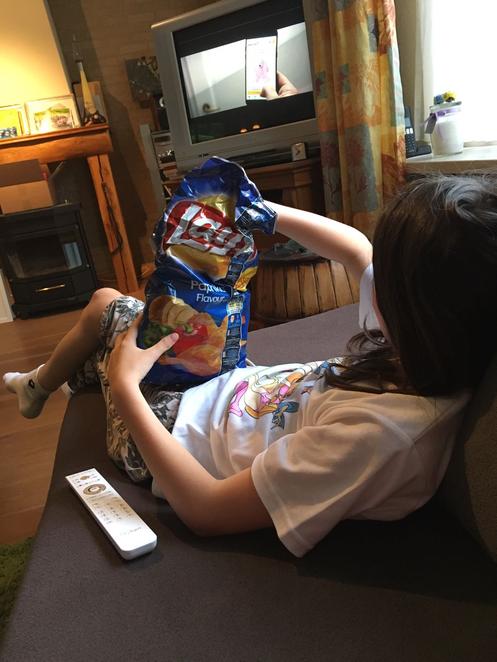

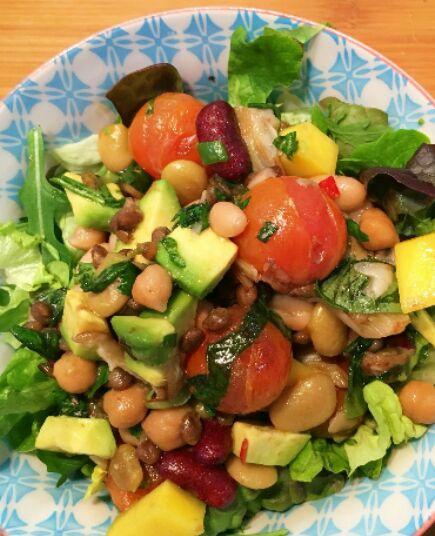

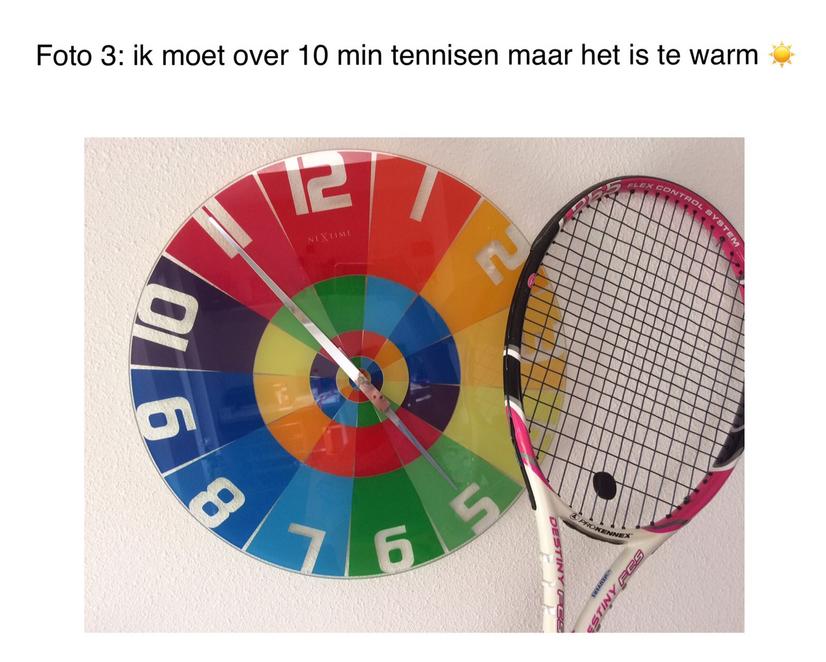

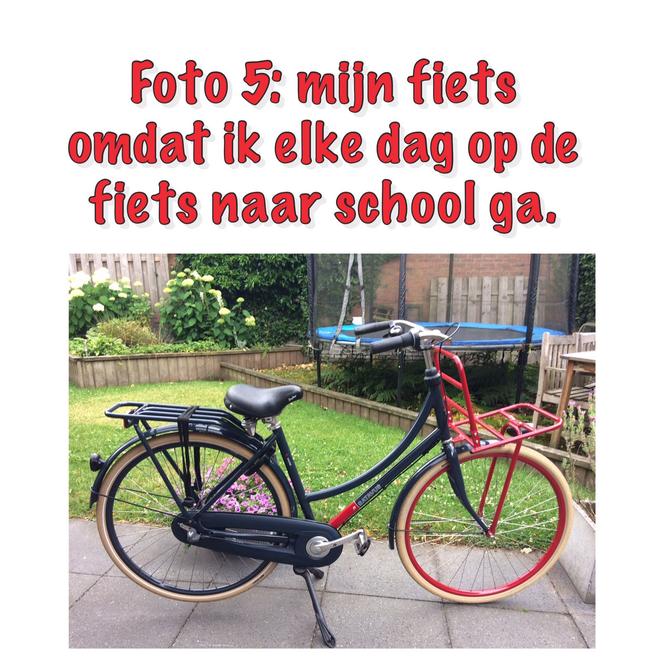

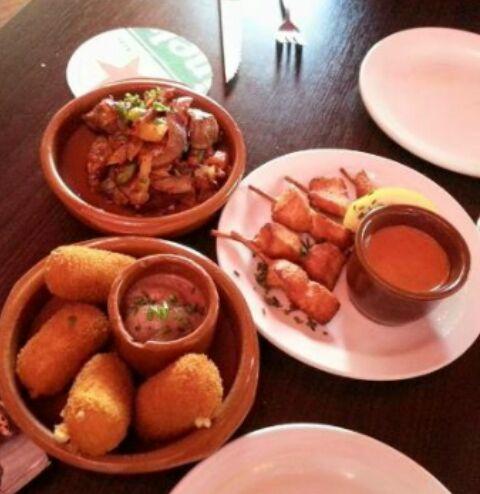
**

**School 2**

**
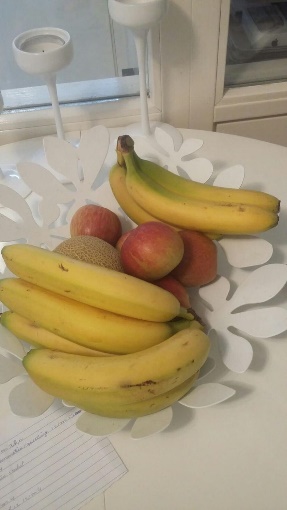

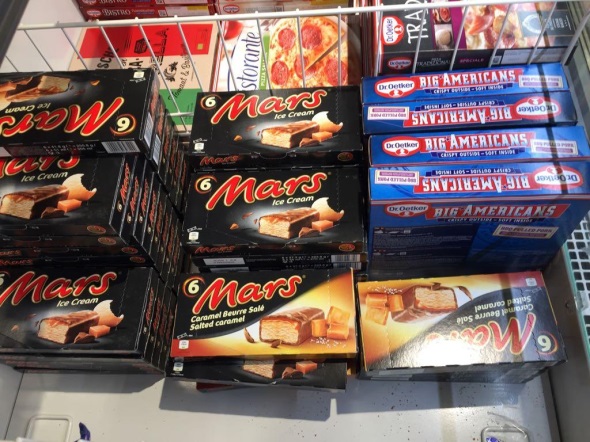

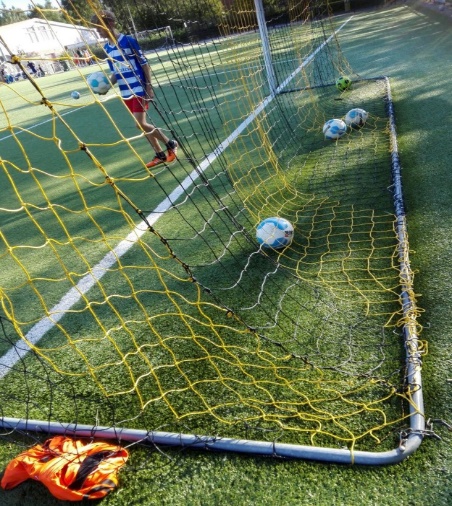

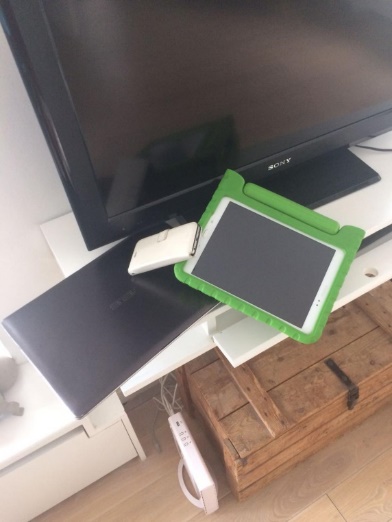

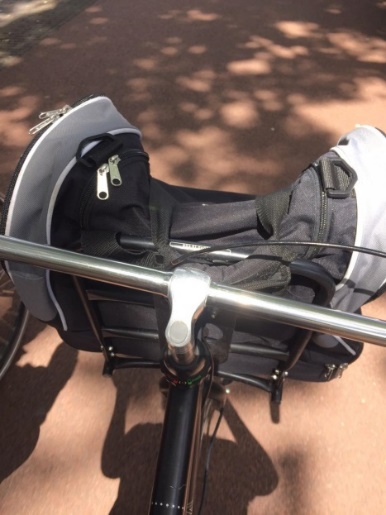
**

**School 3**

**
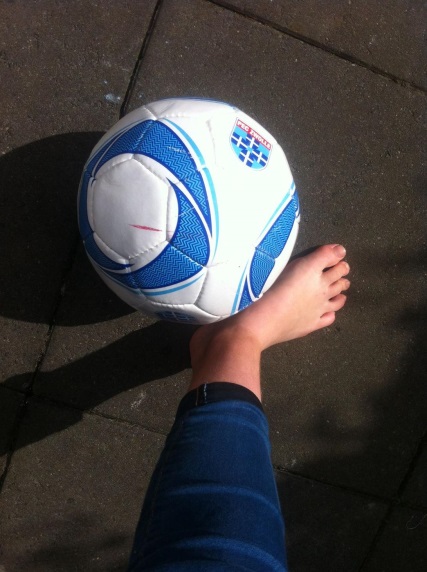

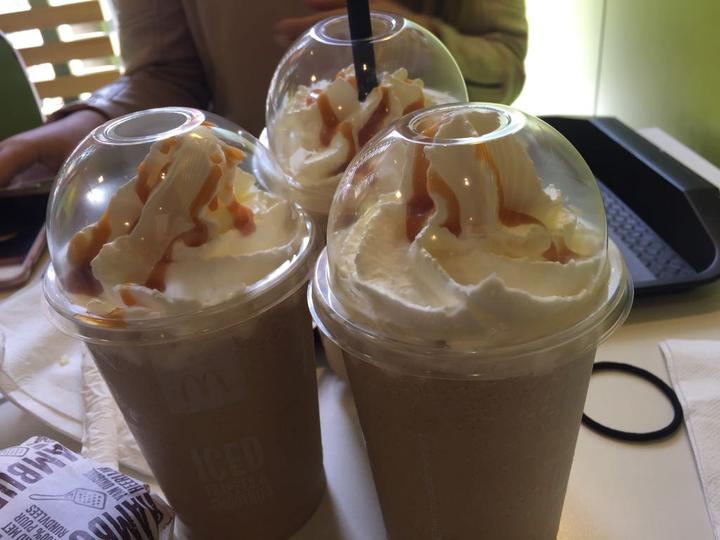
**

**
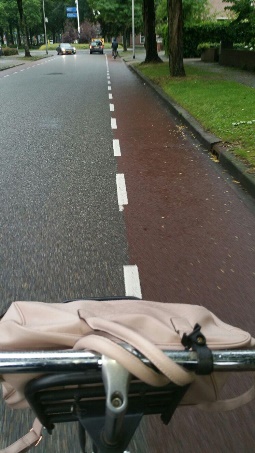

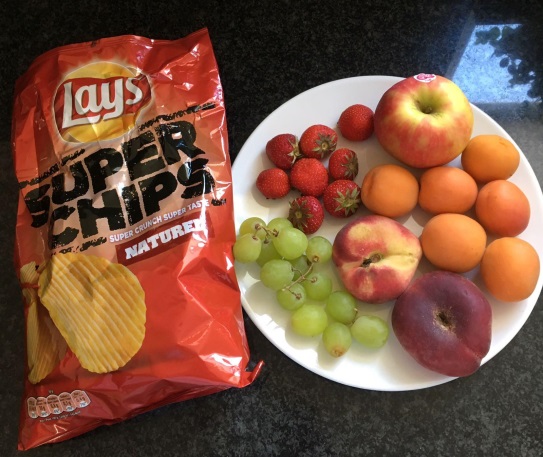

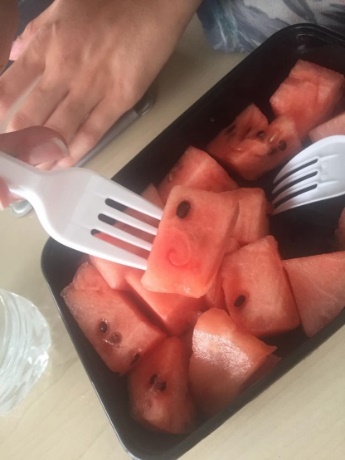
**

**School 4**

**
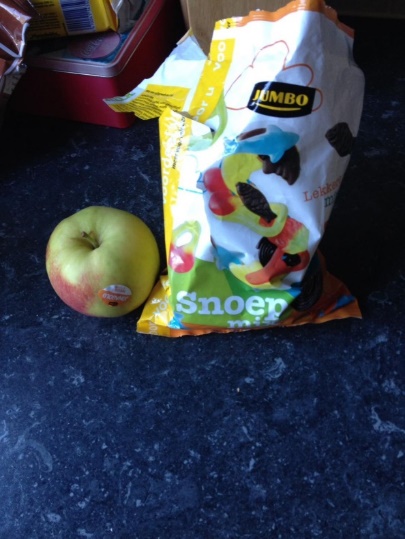

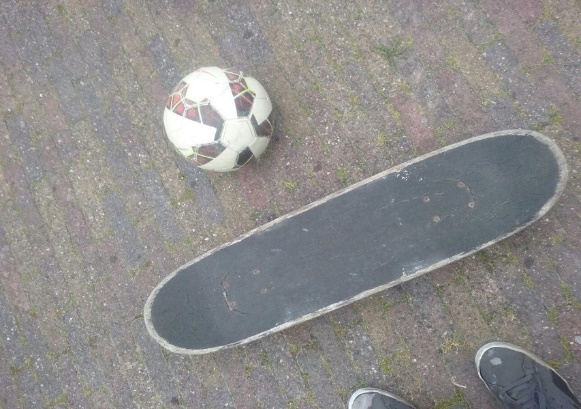

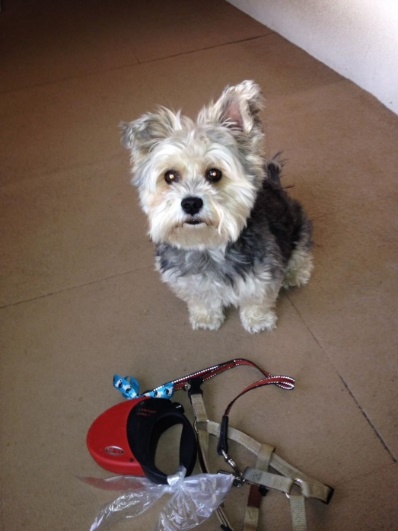

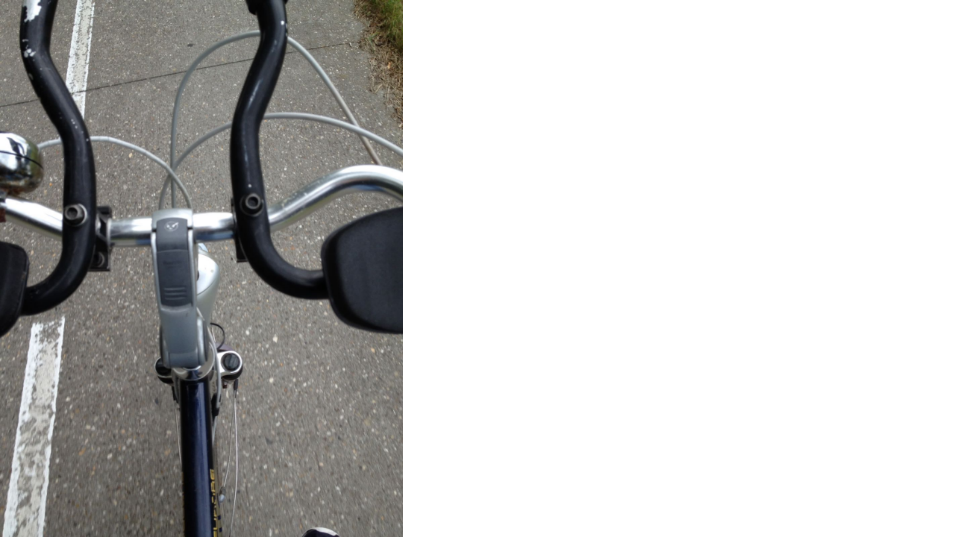

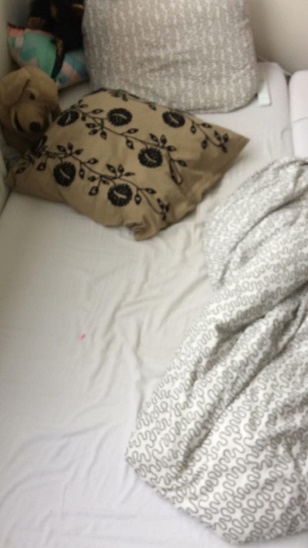
**

Supplement: Supplementary file 1 [file Table_1.DOCX]
